# Umbilical cord-derived Wharton’s jelly for regenerative medicine applications in orthopedic surgery: a systematic review protocol

**DOI:** 10.1186/s13018-020-02067-w

**Published:** 2020-11-11

**Authors:** Benjamin J. Main, Josiah A. Valk, Nicola Maffulli, Hugo C. Rodriguez, Manu Gupta, Ian W. Stone, Saadiq F. El-Amin, Ashim Gupta

**Affiliations:** 1 Beaumont Hospital Farmington Hills, Farmington Hills, MI USA; 2 Department of Musculoskeletal Disorders, School of Medicine and Surgery, University of Salerno, Fisciano, Italy; 3 San Giovanni di Dio e Ruggi D’Aragona Hospital “Clinica Orthopedica” Department, Hospital of Salerno, Salerno, Italy; 4 Queen Mary University of London, Barts and the London School of Medicine and Dentistry, Centre for Sports and Exercise Medicine, London, England; 5 School of Osteopathic Medicine, University of the Incarnate Word, San Antonio, TX USA; 6 South Texas Orthopaedic Research Institute, Laredo, TX USA; 7 Future Biologics, Lawrenceville, GA USA; 8 El-Amin Orthopaedic and Sports Medicine Institute, Duluth, GA USA; 9 BioIntegrate Inc., 2505 Newpoint Pkwy, Suite 100-A, Lawrenceville, GA 30043 USA

**Keywords:** Regenerative medicine, Musculoskeletal injuries, Umbilical cord, Wharton’s jelly, PRISMA

## Abstract

**Background:**

Musculoskeletal injuries and conditions affect millions of individuals. These ailments are typically managed by immobilization, physiotherapy, or activity modification. Regenerative medicine has experienced tremendous growth in the past decades, especially in musculoskeletal medicine. Umbilical cord-derived Wharton’s jelly is an exciting new option for such therapies. Wharton’s jelly is a connective tissue located within the umbilical cord largely composed of mesenchymal stem cells and extracellular matrix components, including collagen, chondroitin sulfate, hyaluronic acid, and sulfated proteoglycans. Wharton’s jelly is a promising and applicable biologic source for orthopedic regenerative application.

**Methods:**

A systematic search will be conducted in PubMed, ScienceDirect, and Google Scholar databases of English, Italian, French, Spanish, and Portuguese language articles published to date. References will be screened and assessed for eligibility by two independent reviewers as per PRISMA guidelines. Articles will be considered without exclusion to sex, activity, or age. Studies will be included if they used culture-expanded, mesenchymal stem/stromal cells of mesenchymal stem cells and/or connective tissue obtained from Wharton’s jelly. Studies will be excluded if Wharton’s jelly is not the sole experimental examined cell type. Placebos, conventional non-operative therapies including steroid injections, exercise, and NSAIDs will be compared. The study selection process will be performed independently by two reviewers using a reference software. Data synthesis and meta-analysis will be performed separately for clinical and pre-clinical studies.

**Discussion:**

The results will be published in relevant peer-reviewed scientific journals. Investigators will present results at national or international conferences.

**Trial registration:**

The protocol was registered on PROSPERO international prospective register of systematic reviews prior to commencement, CRD42020182487.

## Background

Musculoskeletal conditions affect millions of individuals each year and represent a large burden on healthcare. These ailments are classically managed with immobilization, activity modification, physical therapy, and pharmacological agents, and surgery when conservative management has failed. These modalities are limited, often attempting to limit pain rather than addressing the actual pathology [1–7].

Regenerative medicine has undergone tremendous growth during the past few decades, especially in musculoskeletal medicine [8]. Human stem cells offer regenerative potential, aiming to hasten or reverse chronic disease in order to restore function [9–11]. Common sources of metabolically available human stem cells include bone marrow concentrate (BMC), adipose tissue, amniotic tissue, umbilical cord blood, and umbilical cord-derived Wharton’s jelly [12–17]. Stem cell biologic therapies for the treatment of musculoskeletal injuries are becoming more common in orthopedic clinical practice. Increased patient awareness has led to increased demand for biologic therapies [8].

To date, there is no consensus regarding the most appropriate stem cell source. BMC, PRP, and adipose-derived stem cells (ADSCs) have received increased scientific attention given their unique properties and clinical availability. Direct injection therapy has proven beneficial in a human model. Bone marrow mesenchymal stem cells (BMSCs) have limitations associated with increased pain and morbidity from the bone marrow aspiration procedure [18]. BMC has a limited number of BMSCs, with roughly 0.001–0.01% of BMSCs within the BMC [18]. The limited availability and the fact that BMSCs show signs of early senescence urge to identify and exploit other sources of stem cells [18]. The literature on ADSCs is limited, with the lack of randomized double-blinded trials and lack of long-term follow-up observation being the major contributors [19]. The long-term safety of ADSCs is also yet to be determined [19]. PRP has been extensively studied, but most of the results are biased, and from poorly designed studies that favor the publication of positive results [20]. Although safe and promising, PRP still has not shown strong evidence of efficacy and effectiveness in the human clinical setting [20].

Wharton’s jelly is a connective tissue located within the umbilical cord largely composed of hyaluronic acid and chondroitin sulfate. The chief role of Wharton’s jelly is to resist torsional and compressive stresses imposed upon the umbilical vessels during fetal development. Within the Wharton’s jelly are primitive mesenchymal stem cells [21]. These perinatal mesenchymal stem cells resemble embryonic stem cells, but retain many properties of adult mesenchymal stem cells. Wharton’s jelly-derived mesenchymal stem cells express lower levels of pluripotent markers than embryonic stem cells, suggesting they are highly multipotent rather than pluripotent [22, 23]. Wharton’s jelly contains the highest concentration of mesenchymal stem cells per milliliter compared to other tissues with rich extracellular matrix components, including collagen, chondroitin sulfate, hyaluronic acid, and sulfated proteoglycans [24, 25]. Wharton’s jelly has also a clinically relevant quantity of growth factors, cytokines, and extracellular vesicles [1]. The large amount of these substances may play a role in reducing inflammation, pain, and augment healing of musculoskeletal injuries [1].

Wharton’s jelly is easily accessible and available as opposed to other biologics. Every birth presents an opportunity to harvest these highly multipotent, nutrient-rich mesenchymal stem cells. The ease of collection offers many advantages over current BMC and adipose-derived stem cell harvest, which pose donor site morbidity. This factor, as well as the attractive expansion properties of umbilical-derived stem cells and clinically significant amounts of applicable regenerative substances, makes Wharton’s jelly a promising source of mesenchymal stem cells for orthopedic regenerative application [1, 26]. Considering the lack of clarity regarding the use of Wharton’s jelly, methodological factors, clinical translation, and outcome measurement, a systematic review is required to synthesize and evaluate the quality of the available evidence regarding the safety and efficacy of Wharton’s jelly for regenerative medicine applications in orthopedic surgery. The primary objective of this review is to report the clinical, structural, and functional outcomes of the applications of Wharton’s jelly for regenerative medicine in orthopedic surgery. The secondary objective is to identify methodological characteristics associated with application outcomes.

## Methods

The protocol was registered on the PROSPERO international prospective register of systematic reviews, registration number CRD42020182487. The systematic review will follow the Preferred Reporting Items for Systematic Reviews and Meta-Analyses (PRISMA) statement and guidelines [27, 28].

### Eligibility criteria

The PICOS (Population, Intervention, Comparison, Outcome, Study Design) framework will be used as a template for defining eligibility criteria for literature search [29]. Characteristics for clinical studies are as follows.

#### Population

Research involving animal and human models (clinical studies as well as in vitro research) will be considered for review, without exclusion relating to sex or age. Basic science studies must include human cells isolated from a living model. Studies meeting eligibility criteria will apply to acute orthopedic musculoskeletal injury, chronic orthopedic musculoskeletal conditions, or an artificial disease model. Articles will be excluded if they do not relate to orthopedic intervention.

#### Intervention

Research meeting inclusion criteria will involve the use of mesenchymal stem cells and/or connective tissue obtained from Wharton’s jelly. Studies will be excluded if Wharton’s jelly is not the experimental examined cell type. Studies involving umbilical-derived stem cells will be excluded unless the study specifies the use of Wharton’s jelly. Studies will be excluded if they report the use of Wharton’s jelly-derived mesenchymal stem cells in combination with other cell populations.

#### Comparison

Comparators considered will include placebos, non-injury models, acute injury models, non-injury models, and gold standard treatments for orthopedic injury.

#### Outcomes

For basic scientific research, studies relating to human musculoskeletal injury via histological or biochemical measures will be included. For clinical research, studies pertaining to human or animal orthopedic musculoskeletal injury via histological and/or biochemical measures and functional scores (pain, activity, quality of life, etc.) will be included.

#### Study design

Observational studies (cohort, cross-sectional, and case-controlled prospective or retrospective studies) or randomized controlled trials comparing outcomes of connective tissue derived from Wharton’s jelly with control, experimental therapy, or gold standard treatment at any follow-up period will be included. Systematic reviews will only be examined to identify further studies for inclusion, and results of meta-analysis will not be included in the analysis. With regard to publication year, all studies published to date will be included.

### Information sources

A systematic search will be conducted in PubMed, ScienceDirect, and Google Scholar databases of English, Italian, French, Spanish, and Portuguese language articles published before May 2020. Secondary searching of reference lists of key articles and reviews will be undertaken to identify any additional studies potentially missed in electronic search.

### Search

The search and selection process will be based on the PRISMA checklist and flow diagram based on the eligibility and inclusion criteria previously outlined. A web-based reference software system (RefWorks) will be used for data management.

### Study selection

The study selection process will be performed independently by two reviewers. Screening of abstracts will be performed, and full-text articles will be retrieved and uploaded to the reference software. A thorough secondary screening will be performed independently by two reviewers. The secondary screening of the full-text articles will eliminate studies that do not meet inclusion criteria.

### Data collection

Data extraction from articles that meet inclusion criteria will be performed by two independent reviewers. Data extracted and synthesized will include authors, publication year, study design, group controls, group interventions, outcome measurement, and outcome assessment. Customized forms will be used in the data extraction and collection process. The primary authors will be contacted via email for any information necessitating clarification.

### Data items

Relevant items of population, problem, intervention, comparison, and outcome will be extracted and included. For basic scientific research, relevant histological and/or biochemical measures will be included. For clinical research, all histological measures, biochemical measures, and functional scores will be included.

### Risk of bias

Multiple tools will be used to assess the risk of bias for included studies. The Systematic Review Centre for Laboratory Animal Experimentation (SYRCLE) risk of bias tool will be applied to animal studies [30]. Ten domains will be addressed related to selection bias, performance bias, detection bias, attrition bias, reporting bias, and other biases. The Risk Of Bias In Non-randomized Studies of Interventions (ROBINS-I) tool will be used to assess observational and quasi-randomized studies [31]. Seven domains will be utilized to assess risk including confounding, participant selection bias, classification bias, deviation bias, bias due to missing data, outcome measurement bias, and bias in selection of reported results. Studies will be judged to have no information or a low, moderate, serious, or critical risk of bias. For randomized control trials, the Risk of Bias 2 (RoB 2) tool will be used to establish risk of bias [32]. Five domains including biases arising from the randomization process, due to deviations from intended interventions, due to missing outcome data, in measurement of the outcome, and in selection of the reported result will be analyzed. Overall risk of bias will be determined to be low, some concerns, or high. All included studies will be independently scored by two reviewers, and consensus reached by discussion.

### Data synthesis and meta-analysis

Data synthesis and meta-analysis will be performed separately for clinical and pre-clinical studies, following the guidelines published by Hooijmans et al. [30]. Given the sparsity of homogenous research, a qualitative analysis of common outcome variables will be conducted. Subgroups chosen for analysis will include cells derived from Wharton’s jelly versus experimental therapy and/or control. Results of meta-analyses extracted data will be summarized in tables and narrative interpretation provided, with emphasis on outcome measures.

## Discussion

The results of this review will be published in a relevant scientific journal or presented at national or international conferences (“Publications”) by the Investigators.

## Documenting protocol amendments

Protocol amendments and updates will be documented via PROSPERO online register. The nature of the changes made will be recorded, dated, and accessible along with the most recent version within the record audit trail under the systematic review protocol registration number CRD42020182487.

## Data Availability

The results of this review will be published in a relevant scientific journal or presented at national or international conferences (“Publications”) by the Investigators.

## Abbreviations

ADSCs: Adipose-derived stem cells
BMC: Bone marrow concentrate
BMSCs: Bone marrow mesenchymal stem cells
PICOS: Population, Intervention, Comparison, Outcome, and Study Design
PRISMA: Preferred Reporting Items for Systematic Reviews and Meta-Analyses
PRP: Platelet-rich plasma
ROBINS-I: Risk Of Bias in Non-randomized Studies of Interventions
SYRCLE: Systematic Review Centre for Laboratory Animal Experimentation

## References

[1] Gupta A, El-Armin SF, Levy HJ, Sze-Tu R, Ibim SE, Maffulli N (2020). Umbilical cord-derived Wharton’s jelly for regenerative medicine applications. J Orthop Surg Res.

[2] Navani A, Manchikanti L, Albers SL, Latchaw RE, Sanapati J, Kaye AD (2019). Responsible, safe, and effective use of biologics in management of low back pain: American Society of Interventional Pain Physicians (ASIPP) guidelines. Pain Physician.

[3] Gupta A, Woods MD, Illingworth KD, Niemeier R, Schafer I, Cady C (2013). Single walled carbon nanotube composites for bone tissue engineering. J Orthop Res.

[4] Gupta A, Main BJ, Taylor BL, Gupta M, Whitworth CA, Cady C (2014). In vitro evaluation of three-dimensional single-walled carbon nanotube composites for bone tissue engineering. J Biomed Mater Res A.

[5] Gupta A, Liberati TA, Verhulst SJ, Main BJ, Roberts MH, Potty AG (2015). Biocompatibility of single-walled carbon nanotube composites for bone regeneration. Bone Joint Res.

[6] Vallejo R, Gupta A, Kelly CA, Vallejo A, Rink J, Williams JM (2020). Effects of phase polarity and charge balance spinal cord stimulation on behavior and gene expression in a rat model of neuropathic pain. Neuromodulation..

[7] Gupta A, Sharif K, Walters M, Woods MD, Potty A, Main BJ, et al. Surgical retrieval, isolation and in vitro expansion of human anterior cruciate ligament-derived cells for tissue engineering applications. J Vis Exp. 2014;86. 10.3791/51597 PMC4184405 24836540

[8] Lamplot JD, Rodeo SA, Brophy RH (2020). A practical guide for the current use of biologic therapies in sports medicine. Am J Sports Med.

[9] Caplan AI (1991). Mesenchymal stem cells. J Orthop Res.

[10] Minguell JJ, Erices A, Conget P (2001). Exp Biol Med (Maywood).

[11] Andia I, Maffulli N (2019). New biotechnologies for musculoskeletal injuries. Surgeon..

[12] Le ADK, Enweze L, DeBaun MR, Dragoo JL (2018). Current clinical recommendations for use of platelet-rich plasma. Curr Rev Musculoskelet Med.

[13] Patel JM, Saleh KS, Burdick JA, Mauck RL (2019). Bioactive factors for cartilage repair and regenerations: improving delivery, retention, and activity. Acta Biomater.

[14] Le ADK, Enweze L, DeBaun MR, Dragoo JL (2019). Platelet-rich plasma. Clin Sports Med.

[15] Sezgin EA, Atik OS (2018). Are orthobiologics the next chapter in clinical orthopedics? A literature review. Eklem Hastalik Cerrahisi.

[16] Duerr RA, Ackermann J, Gomoll AH (2019). Amniotic-derived treatments and formulations. Clin Sports Med.

[17] Chirichella PS, Jpw S, Iacono S, Wey HE, Malanga GA (2019). Treatment of knee meniscus pathology: rehabilitation, surgery, and orthobiologics. PM R.

[18] Mohamed-Ahmed S, Fristad I, Lie SA, Suliman S, Mustafa K, Vindenes H, et al. Adipose-derived and bone marrow mesenchymal stem cells: a donor-match comparison. Stem Cell Res Ther. Stem Cell Res Ther. 2018;9(1):168. 10.1186/s13287-018-0914-1 PMC6008936 29921311

[19] Usuelli FG, D’Ambrosi R, Maccario C, Indino C, Manzi L, Maffulli N (2017). Adipose-derived stem cells in orthopaedic pathologies. Br Med Bull.

[20] Brossi PM, Moreira JJ, Machado TS, Baccarin RY (2015). Platelet-rich plasma in orthopedic therapy: a comparative systematic review of clinical and experimental data in equine and human musculoskeletal lesions. BMC Vet Res.

[21] Troyer DL, Weiss ML (2008). Concise review: Wharton’s jelly-derived cells are a primitive stromal cell population. Stem Cells.

[22] Carlin R, Davis D, Weiss M, Schultz B, Troyer D (2006). Expression of early transcription factors Oct-4, Sox-2 and Nanog by porcine umbilical cord (PUC) matrix cells. Reprod Biol Endocrinol.

[23] La Rocca G, Anzalone R, Corrao S, Magno F, Loria T, Lo Iacono M (2009). Isolation and characterization of Oct-4þ/HLA-Gþ mesenchymal stem cells from human umbilical cord matrix: differentiation potential and detection of new markers. Histochem Cell Biol.

[24] Vangsness CT, Sternberg H, Harris L (2015). Umbilical cord tissue offers the greatest number of harvestable mesenchymal stem cells for research and clinical application: a literature review of different harvest sites. Arthroscopy..

[25] Sobolewski K, Małkowski A, Bańkowski E, Jaworski S (2005). Wharton’s jelly as a reservoir of peptide growth factors. Placenta..

[26] Schugar RC, Chirieleison SM, Wescoe KE, Schmidt BT, Askew Y, Nance JJ, et al. High harvest yield, high expansion, and phenotype stability of CD146 mesenchymal stromal cells from whole primitive human umbilical cord tissue. J Biomed Biotechnol. 2009. Article ID 789526:11. https://www.hindawi.com/journals/bmri/2009/789526/. 10.1155/2009/789526 PMC2796378 20037738

[27] Moher D, Liberati A, Tetzlaff J, Altman DG, The PRISMA Group (2009). Preferred Reporting Items for Systematic Reviews and Meta-Analyses: the PRISMA statement. Open Med; 3(3); 123-130. PMC3090117 21603045

[28] Liberati A, Altman DG, Tetzlaff J, Mulrow C, Gøtzsche PC (2009). The PRISMA statement for reporting systematic reviews and meta-analyses of studies that evaluate health care interventions: explanation and elaboration. BMJ.

[29] O’Connor D, Green S, Higgins J (2008). Defining the review question and developing criteria for including studies, Cochrane handbook for systematic reviews of interventions: Cochrane book series.

[30] Hooijmans CR, Rovers MM, de Vries RB, Leenaars M, Ritskes-Hoitinga M, Langendam MW (2014). SYRCLE’s risk of bias tool for animal studies. BMC Med Res Methodol.

[31] Sterne JA, Hernán MA, Reeves BC, Savović J, Berkman ND, Viswanathan M (2016). ROBINS-I: a tool for assessing risk of bias in non-randomized studies of interventions. BMJ..

[32] Sterne JAC, Savović J, Paige MJ, Elbers RG, Blencowe NS, Boutron I (2019). RoB 2 a Revised tool for assessing risk of bias in randomised trials. BMJ..

